# A transwell assay that excludes exosomes for assessment of tunneling nanotube-mediated intercellular communication

**DOI:** 10.1186/s12964-017-0201-2

**Published:** 2017-11-13

**Authors:** Venugopal Thayanithy, Patrick O’Hare, Phillip Wong, Xianda Zhao, Clifford J. Steer, Subbaya Subramanian, Emil Lou

**Affiliations:** 1 Department of Medicine, Division of Hematology, Oncology and Transplantation, University of Minnesota, Mayo Mail Code 480, 420 Delaware Street SE, Minneapolis, MN 55455 USA; 2 Department of Surgery, University of Minnesota, Minneapolis, MN 55455 USA; 3 Department of Medicine, Division of Gastroenterology, Hepatology and Nutrition, University of Minnesota, Minneapolis, MN 55455 USA; 4 Department of Genetics, Cell Biology and Development, University of Minnesota, Minneapolis, MN 55455 USA; 5 Graduate Faculty, Department of Integrative Biology and Physiology, University of Minnesota, Minneapolis, MN 55455 USA; 6 Present Address: Molecular Diagnostics Laboratory, University of Minnesota Medical Center, Fairview, 420 Delaware St SE, MMC 198, Minneapolis, MN 55455 USA

**Keywords:** Tunneling nanotubes, Membrane nanotubes, Intercellular transfer, Intercellular communication, Transwell assay, Exosomes, Microvesicles, Extracellular vesicles

## Abstract

**Background:**

Tunneling nanotubes (TNTs) are naturally-occurring filamentous actin-based membranous extensions that form across a wide spectrum of mammalian cell types to facilitate long-range intercellular communication. Valid assays are needed to accurately assess the downstream effects of TNT-mediated transfer of cellular signals in vitro. We recently reported a modified transwell assay system designed to test the effects of intercellular transfer of a therapeutic oncolytic virus, and viral-activated drugs, between cells via TNTs. The objective of the current study was to demonstrate validation of this in vitro approach as a new method for effectively excluding diffusible forms of long- and close-range intercellular transfer of intracytoplasmic cargo, including exosomes/microvesicles and gap junctions in order to isolate TNT-selective cell communication.

**Methods:**

We designed several steps to effectively reduce or eliminate diffusion and long-range transfer via these extracellular vesicles, and used Nanoparticle Tracking Analysis to quantify exosomes following implementation of these steps.

**Results:**

The experimental approach outlined here effectively reduced exosome trafficking by >95%; further use of heparin to block exosome uptake by putative recipient cells further impeded transfer of these extracellular vesicles.

**Conclusions:**

This validated assay incorporates several steps that can be taken to quantifiably control for extracellular vesicles in order to perform studies focused on TNT-selective communication.

**Electronic supplementary material:**

The online version of this article (10.1186/s12964-017-0201-2) contains supplementary material, which is available to authorized users.

## Background

Intercellular communication is an important biological process that was originally thought to occur exclusively via cytokine, chemokine, hormone, and growth factor signaling, in addition to intercellular transfer of signals via gap junctions. During the past decade, the discovery of exosomes, microvesicles (MV), and long actin-based membranous cellular extensions termed “tunneling nanotubes” (TNTs) has significantly expanded the repertoire of known mediators of intercellular signaling.

TNTs are structures that can extend up to several hundreds of microns between cells to provide direct conduits of communication. The width of TNTs can range from 50 to 1000 nm, and their length ranges from a few to several hundred microns. Several dozen published studies have incorporated use of transwell assays to study effects of long-distance intercellular communication, either by TNTs or by exosomes/microvesicles [1–26] (summarized in Tables 1 and 2). However, the results of these studies have been difficult to interpret for several reasons. First, many of these studies assumed that TNTs are not capable of traversing the membrane filter and facilitating intercellular transfer between separated cell populations. Thus they used standard transwell/Boyden chamber assays as negative controls with the intent of demonstrating lack of TNT communication. Second, and conversely, protocols that applied the same in vitro system to evaluate TNT-mediated transfer did not include specific measures to identify the effects of exosomes and microvesicles (herewith referred to collectively as extracellular vesicles, or EVs) from the effects of TNTs. Identification and validation of such an assay is imperative in moving this field of cell communication forward. We postulated that adding a series of steps to modify the standard transwell assay would effectively reduce or even eliminate intercellular trafficking of EVs, and thus negate any potential downstream effects of these EVs that could confound investigation of the effects of TNT-mediated transport.

**Table 1:** Published studies using transwell membranes for investigation of cell contact-dependent intercellular transfer

Purpose of experiment	Pore size of the transwell filter	Reference	Effect of the transwell filter	Observations/Conclusions
Transfer of HIV-1 virions between Jurkat T cells	3000 nm	[14] Sowinski et al., 2008	Negative	Decreased HIV-1 transfer
Transfer of apoptotic signal from stromal cells to B cells	3000 nm	[15] Teague et al., 2010	Negative	Cell contact was necessary for transfer
Transfer of miRNAs between cancer and endothelial cells	3000 nm & 400 nm	[2] Connor et al., 2015	Negative	Transfer of miRNA was reduced to basal levels
Lysosomes mediated transfer of cystinosin & cystinebetween mouse fibroblasts and macrophages	1000 nm	[7] Naphade et al., 2015	Negative	Decreased cysteine transfer
Transfer of DiD between NRK, CHO and HeLa cells	450 nm	[13] Schoelermann et al., 2015	Negative	Transfer of DiD was reduced
Transfer of CD40L-induced cytoplasmic and cell surface–associated material from T cells to dendritic cells	450 nm	[17] Zaccard et al., 2014	Negative	Decrease in transfer
Transfer of prion particles between mouse neuronal cells	400 nm	[4] Gousset et al., 2009	Negative	Prion transfer was entirely abolished
Transfer of cues for cell migration from endothelial cells to bronchial epithelial cells	400 nm	[18] Zani et al., 2010	Negative	Bronchial epithelial cell migration required direct cell contact with endothelial cells
Transfer of prion particles in mouse neuronal cells	400 nm	[5] Langevin et al., 2010	Negative	Prion transfer was reduced over 98%
Transfer of mitochondria from vascular smooth muscle cells to mesenchymal cells	400 nm	[16] Vallabhaneni et al., 2012	Negative	Direct cell contact was required for mitochondrial transfer
Transfer of p-glycoprotein between MCF-7 breast cancer cells	400 nm	[42] Pasquier et al., 2012	Negative	Reduced p-glycoprotein transfer
Transfer of Huntington mutant protein (Htt) aggregates in mouse neuronal cells	400 nm	[3] Costanzo et al., 2013	Negative	Htt aggregate transfer was blocked by over 95%
Transfer of contact dependent proliferation cues from astrocytes to glioma cells	400 nm	[19] Zhang and Zhang, 2015	Negative	Decreased transfer of molecular cues
Transfer of mCherry from senescent cells to NK cells	400 nm	[1] Biran et al., 2015	Negative	mCherry transfer was reduced
Transfer of soluble amino acids between bacterial cells	200 nm	[9] Pande et al., 2015	Negative	Amino acid transfer was entirely blocked
Mitochondrial transfer in MDA-MB231, OVCAR3, SKOV3 and MCF-7 cells	NA	[42] Pasquier et al., 2013	Negative	Decrease in mitochondrial transfer

These experiments used transwell membrane filters with a range of pore sizes to separate two populations of cells, in order to demonstrate cellular transfer that was dependent on cell contact by any means (including TNTs)

**Table 2:** Published studies using transwell assays to investigate EV-specific intercellular transfer

Purpose of experiment	Pore size of the transwell filter	Reference	Effect of the transwell filter on EV transfer	Observations/Conclusions
Transfer P-glycoprotein between cells from human bladder cancer cell line BIU-87	3000 nm	[26] Zhou et al., 2013	Neutral	Transfer was not affected; p-glycoprotein transfer does not require direct cell-to-cell contact
Transfer of HIV-1 viral particles between T cells	3000 nm	[6] Martin et al., 2010	Neutral	Transwell filter allowed HIV-1 viral transfer
Transfer of exosomes from oligodendrocyte to primary cortical neurons	1000 nm	[46] Fruhbeis et al., 2013	Neutral	Transwell filter allowed exosome transfer
Exosome-mediated transfer of EBV-encoded small RNAs between lymphoblasts and dendritic cells	1000 & 400 nm	[20] Zomer et al., 2010	Negative	Decreased small RNA transfer
Transfer of membrane proteins between Chinese hamster ovary (CHO) cells in culture as well as via 200 nm filtered culture media	450 nm & 200 nm	[8] Niu et al., 2008	Negative	Reduced transfer of membrane proteins
Exosome-mediated transfer of Cy3-labeled microRNAs from leukemia cells to endothelial cells	450 nm	[25] Umezu et al., 2013	Neutral	Transwell filter allowed transfer of miRNAs
Exosome-mediated transfer of Cy3-labeled synthetic 22 bp miRNA, from Burkitt lymphoma B cells to T cells	400 nm	[12] Rechavi et al., 2009	Negative	miRNA transfer was reduced
Transfer of TGFβ-1 from platelets to colon and breast carcinoma cells	400 nm	[23] Labelle et al., 2011	Neutral	Transwell filter allowed transfer
Exosome-mediated transfer of G-protein coupled receptors in U87MG, COS-7 or HEK293T cells	400 nm	[21] Guescini et al., 2012	Neutral	Transwell filter allowed TGFβ-1 transfer
Transfer of anti-miR-9 from MSCs to glioblastoma cells	400 nm	[24] Munoz et al., 2013	Negative	Transfer was reduced from 90% to 16%
Transfer of secretory factors from mesenchymal stem cells to nucleus pulposus cells	400 nm	[22] Hu et al., 2015	Neutral	Transwell filter allowed transfer of secretory factors

## Methods

### Cell lines and culture medium

MSTO-211H (MSTO) is derived from a patient with biphasic mesothelioma (ATCC no. CRL-2081). VAMT is a sarcomatoid mesothelioma cell line. Both cell lines were obtained courtesy of Dr. Yuman Fong, Memorial Sloan-Kettering Cancer Center. All cell lines were passaged in plasmocin-containing medium (Invivogen, San Diego, CA) and tested negative for mycoplasma contamination. Cell lines MSTO-211H and VAMT were authenticated by the Core Fragment Analysis Facility at Johns Hopkins University using short tandem repeat profiling. All cells were passaged using 10% fetal calf serum (FCS) in RPMI-1640 with 25 mM glucose, supplemented with 1% penicillin-streptomycin (P-S) and 2% L-glutamine, at normal pH (7.6). To stimulate nanotube formation for in vitro examination, cells were grown in 2.5% FCS in RPMI-1640 containing 50 mM glucose, supplemented with 1% P-S, 2% L-glutamine with or without 10 mM ammonium lactate (Sigma Aldrich, St. Louis, Missouri) and acidification of medium to pH 6.6, per our prior study [27]. All cultures were done in 75cm^2^ tissue culture flasks (Falcon, Becton Dickson, Oxnard, CA) at 37 °C in 5% CO_2_.

### Imaging of TNTs traversing the polyester membrane filter (modified transwell assay)

MSTO-211H cells were primed in TNT medium for 3 days, after which TNT culture medium was removed and replaced with serum-free mTeSR media containing pituitary bovine extract for 24 more hours. Cells were harvested by trypsinization, washed and stained with fluorescent DiI for 15 min at 37 °C, following standard protocols (Life Technologies, Carlsbad, CA). 8.0 × 10^5^ DiI-stained MSTO cells were placed in the upper chamber of transwell assays containing inserts with 0.4 μm diameter pores, and incubated for 48 h. Of note, the transwell insert consists of a polyester membrane 10.0 μm thick and contains ~ 4 × 10^6^ pores/cm^2^.

After 48 h, culture medium was removed and cells were gently washed twice in ice cold phosphate buffered saline (PBS) and fixed in ice cold neutral buffered formalin (NBF) for 15 min. The polyester membrane was cut from the transwell insert and transferred to a stainless steel mounting chamber (Attofluor cell chamber, Life Technologies, Carlsbad, CA) using two 25 mm round glass coverslips of 0.17 mm thickness (Warner Instruments, Hamden, CT). The membrane was mounted onto a glass slide with Fluorshield (Sigma-Aldrich, St. Louis, MO, catalog number F6182) and imaged on a Nikon A1RMP confocal microscope using a 25× water-immersion lens at laser scanning confocal modality using 561 nm excitation and 595 nm emission. Since the membrane was 10 μm in thickness, imaging data were collected for a total thickness of 30 μm from the middle depth of the membrane. In order to map the spatial and temporal location of the cells on the transwell membrane precisely, we employed transmitted detection (TD). Transmitted images were acquired simultaneously along with confocal images in real time by collecting the signals that were transmitted through the sample using the same illumination beam. Images were analyzed with NIS elements AR software (Nikon, version 4.00.07).

### Isolation of exosomes

Exosomes were isolated using the Total Exosome Isolation Kit following the manufacturer’s protocol (Invitrogen, Carlsbad, CA, Catalog number 4478359) as previously described [28]. Briefly, VAMT cells were grown to confluence in T75 flasks using 5% FCS RPMI for 3 days, and cells were washed with mTeSR1 basal medium (Stemcell Technologies, Vancouver, BC, Canada) 3 times and grown in mTeSR1 basal medium for 2 days prior to exosome isolation. Exosomes were isolated and purified by successive ultracentrifugation steps [29]. Media from VAMT cell cultures (18 ml each) were collected, and (9 ml of each) was added to 15 ml Beckman centrifuge tubes (Beckman Coulter, Brea, CA, catalog number 342082) and spun at 2000 x g for 30 min to remove cells and debris. Supernatant was transferred (9 ml each) into fresh 15 ml centrifuge tubes, and 4.5 ml of exosome isolation reagent was added and mixed by inverting the tube a few times. Exosomes were precipitated by incubating at 4 °C overnight and collected by centrifuging at 10,000 x g for 1.0 h at 4 °C. Supernatant was carefully removed by aspiration. Exosomes were then resuspended in PBS, aliquoted in 100 μl fractions, and stored at −80 °C until use.

### Preparation of cells, transwell membrane incubation, and exosome recovery for imaging of VAMT exosomes using cryo-electron microscopy

To assess the ability of exosomes to traverse the physical barrier of a transwell membrane with the smallest available pore size (0.4 μm), we added VAMT-derived exosomes to MSTO cells adhering to the transwell membrane filter in the the top chamber, as follows:

#### Preparation of MSTO cells

MSTO cells were first grown in 10% FBS RPMI-1640 medium for 24 h. This medium was then removed, and adherent cells in the flasks were washed 3 times with serum-free/vesicle-free mTeSR medium. To minimize the effects of exosome secretion by MSTO cells, this medium was replaced with fresh mTeSR medium after 24 h. Then, the MSTO cells (0.3 × 10^6^) were trypsinized and seeded on to 6-well plates with fresh mTeSR medium in triplicates with or without the transwell inserts each containing the same number of cells. After overnight incubation, the medium was changed and cells were subjected to incubation with VAMT-derived exosomes (0.3 × 10^6^).

#### Transwell incubation

To assess the ability of exosomes to traverse the physical barrier of a transwell membrane, MSTO cells were seeded on the smallest available pore size (0.4 μm) overnight; once the cells adhered to the membrane, we then added VAMT-derived exosomes to the top chamber (0.3 × 10^6^) for incubation over 48 h.

#### Exosome recovery

After 48 h, we collected the culture medium from the bottom wells in order to determine the amount of exosomes that could have passed through the membrane filter during the incubation period. The media collected from the bottom wells (6 ml of media total, from 3 identical wells) with or without addition of exosomes were collected and subjected to exosome isolation [30]. Briefly, the media were transferred to 15 ml Beckman tubes (Beckman Coulter, Indianapolis, IN; catalog number #342082) and centrifuged at 2000 g for 30 min to sediment contaminating cells. Clean cell-free media (6 ml each) were transferred into fresh 15 ml centrifuge tubes and 3 ml of exosome isolation reagent was added (Invitrogen, Carlsbad, CA; catalog number 4478359) and mixed by inverting the tubes three times. Exosomes were precipitated by incubating the mix overnight at 4 °C and collected by centrifuge at 10,000 g for 1 h at 4 °C. Supernatant was removed by aspiration and the exosome pellet was suspended in 100 μl of PBS and stored at −80 °C until use.

### Nanoparticle tracking analysis

Nanoparticle tracking analysis (NTA) operates on the Stokes-Einstein relationship. In short, the phenomenon of size dependence of solute diffusivity of nanoparticles in Brownian motion in a liquid suspension is determined by size, temperature, and viscosity. Briefly, digital images of scattered light from single particles were recorded and plots of scattered light spots and their speed of motion were subjected to software-based analysis to determine the particle count and size distribution.

We used a NanoSight LM14 analyzer (Malvern, Worcestershire, UK), at the University of Minnesota-Twin Cities Nano Center. Samples were diluted in 1.0 ml of PBS (viscosity 0.952–0.953 cP) viewed, background corrected, and subjected to analysis at standard conditions recommended by the manufacturer. Video micrographs of particles undergoing Brownian motion were captured for 60 s at 22 °C using a SCMOS camera from which particle size and distribution was analyzed at recommended settings using NTA software (version 3.0068). Each sample was run 5 times independently, and the average number of particles was included for comparison.

### Scanning electron microscopy of TNTs traversing the polyester membrane

MSTO cells preconditioned in TNT media for 7 days were plated onto transwell inserts containing polyester membranes with 400 nm pores on a 6-well plate (Corning Life Sciences, Corning, NY, Costar product #3450 clear) in regular RPMI-1640 media with 10% FBS, with antibiotics at standard cell culture conditions. After 48 h, medium was removed and the membrane was gently washed with ice cold PBS and fixed with 4% PFA for 10 min and washed gently in ice cold PBS. In order to visualize TNTs traversing the membrane through the 400 nm pores, the opposite side of the transwell membrane was imaged. The membrane was sliced out and mounted on to the SEM stage exposing the bottom side of the membrane. In order to detect TNTs, the membrane was imaged in vacuum without any processing or staining on a Sigma 500 VP FESEM (Carl Zeiss Industrial Metrology, Maple Grove, MN).

### Staining of exosomes

1 × 10^8^ exosomes were stained using 10 μl of PKH26 red fluorescent dye in a total volume of 400 μl at room temperature, as recommended by the vendor (Sigma-Aldrich, St Louis, MO). After 10 min staining was stopped by adding equal volume of 1% BSA, and exosomes were recovered as mentioned using total exosome isolation reagent (Invitrogen).

### Heparin treatment to decrease EV uptake by putative recipient cells in the bottom chamber

MSTO cells were plated in glass-bottomed 6-well plates (MatTek Corporation, Ashland, MA) in cell culture passage medium (10% FBS, RPMI-1640), and cells were allowed to settle overnight. Differences in exosomal uptake were investigated with or without pre-treating the cells with 10 μg/ml of heparin (Heparin sodium salt from porcine intestinal mucosa, Sigma-Aldrich, St Louis, MO) in PBS for 30 minutes [31]. Cells were washed in 2.0 ml of passage medium, and exosomes (100 exosomes/cell) were added in 500 μl fresh medium and allowed to coat the cells by gently tilting the plates three times, followed by 15 min incubation at 37 °C. Exosome-containing medium was removed, cells were washed with passage medium and then returned to the incubator with 2.0 ml of passage culture medium. Exosome uptake was determined by fluorescent microscopic imaging after 24 h. This experiment was performed in triplicate, and representative images were taken from each of the replicates for subsequent analysis. Distribution of fluorescent exosomes was further analyzed using ImageJ software (https://imagej.nih.gov/ij/download.html) to assess the Corrected Total Cell fluorescence (CTCF) of cells (TNT-positive and TNT-negative cells) using the following equation:

CTCF = Integrated Density - (Area of selected cell * Mean fluorescence of the background readings).

CTCF/area was calculated for 36 cells in the control group, and 72 cells in the heparin-treated group, and the results were averaged.

### Availability of materials and data

The datasets generated during and/or analyzed during the current study are included in this published article (and its Additional files 1, 2, 3, 4 and 5), and also are available from the corresponding author on request.

## Results

### Overview of modifications to the transwell assay for assessing TNT-selective intercellular transfer

The use of a transwell assay comprising two culture chambers separated by a porous membrane filter helps to eliminate gap junction-mediated intercellular communication through physical separation (Additional file 1: Figure S1). However, the transwell membrane can still allow the diffusion of EVs between separated populations of cells. To develop a transwell assay that specifically isolates TNT-mediated intercellular communication, it was necessary to address several concerns to more thoroughly exclude EV transfer. First, serum commonly used in culture medium inherently contains exosomes; and when loading serum-containing media to the top well of the assay, these exosomes may be transmitted via normal diffusion to the bottom well. Second, cells themselves, prior to and following deposit into the top chamber, shed EVs, and by so, decreasing this shedding would make exosomal transfer to cells in the bottom chamber less likely. Third, commercially available transwell assays can be selected based on pore size within the membrane filter; and the pore sizes may be as large as 3 or 8 μm. A large pore size can easily permit trafficking of exosomes and larger MVs and microparticles. Larger pore size may also permit cell contorsion and movement from the top to the bottom chamber, giving a false sense of communication due to cell movement itself. The exosomes or other vesicles that cross the filter via the pores might be absorbed by those cells in the bottom chamber, confounding the interpretation of TNT-selective transfer between donor and recipient cells.

For this study, we used cells derived from human malignant pleural mesothelioma as a model system, as we have used these cells extensively in our previously published studies characterizing TNTs. MSTO-211H (also referred to as MSTO) is representative of the biphasic histologic subtype of mesothelioma, and VAMT is derived from the sarcomatoid histologic subtype. These cell lines readily form TNTs in cell culture, and thus are optimal for characterizing these unique cell protrusions [27, 32, 33]. We have previously noted that TNT formation in MSTO and VAMT cells is upregulated in conditions of metabolic and physiological stress, such as a low-serum, hyperglycemic microenvironment [27]; actin is uniformly present throughout the length of these TNTs, and ezrin and ZO-1 localize at the bases of TNTs in these cells; MSTO TNTs facilitate intercellular transfer of molecular cargo such as mitochondria and Golgi vesicles [27, 34]; and formation of TNTs in these cells can be suppressed via inhibition of mTOR. In a subsequent study, we further characterized TNTs in mesothelioma using these specific cell lines, and found that the average rate of formation of TNTs varied based on histopathologic subtype; the average length of MSTO and VAMT TNTs diminished over time [32]; and gene expression profiling of MSTO cells revealed variable regulation of genes responsible for cell cycle regulation, invasion, migration, and metastasis when cells were pre-cultured in low-serum, hyperglycemic medium. We also discovered that exosomes derived from malignant mesothelioma cells induced upregulation of TNT formation between MSTO cells, and that material from these exosomes subsequently shuttled through these newly formed TNTs [30]. Thus, based on our extensive prior characterization of TNTs in MSTO and VAMT cells, we elected to use this model system to develop and examine our transwell assay, but with the idea that the assay could potentially be applied to other TNT-forming cell types (malignant and non-malignant alike) as well.

### Visual evidence of TNT formation and passage through and past the transwell filter using confocal microscopic imaging

**Fig. 1:**
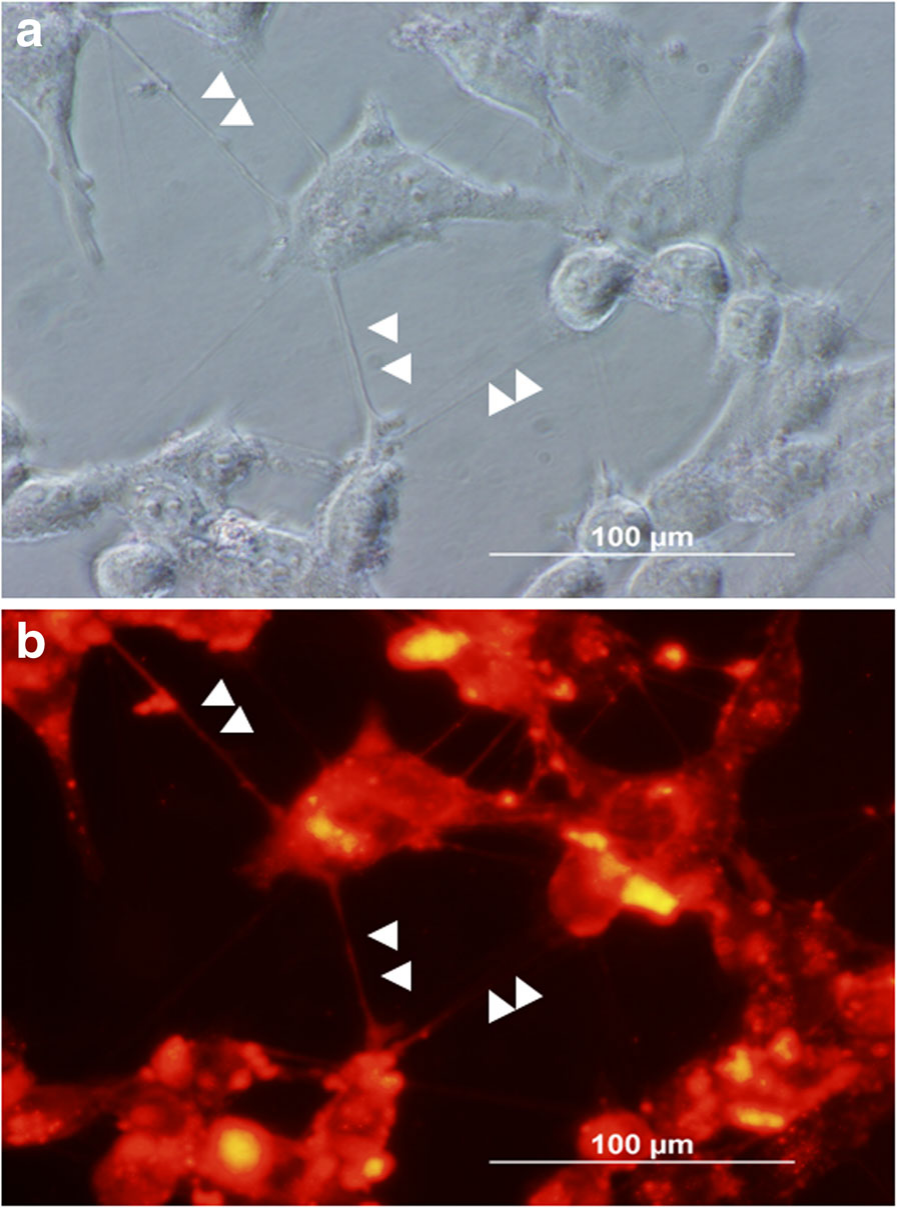
Representative images of TNT formation of MSTO-211H malignant pleural mesothelioma cells in open culture (6-well standard culture plates). TNTs connecting DiI-stained MSTO cells are marked by white arrowheads. MSTO cells were first grown using standard passage medium (10% FCS RPMI-1640), then switched to a low-serum, hyperglycemic RPMI-1640 culture medium, stained with lipophilic DiI, and imaged after 48 h on a Olympus X70 fluorescent microscope at 600× magnification using phase contrast (**a**) and fluorescent (**b**) settings

**Fig. 2:**
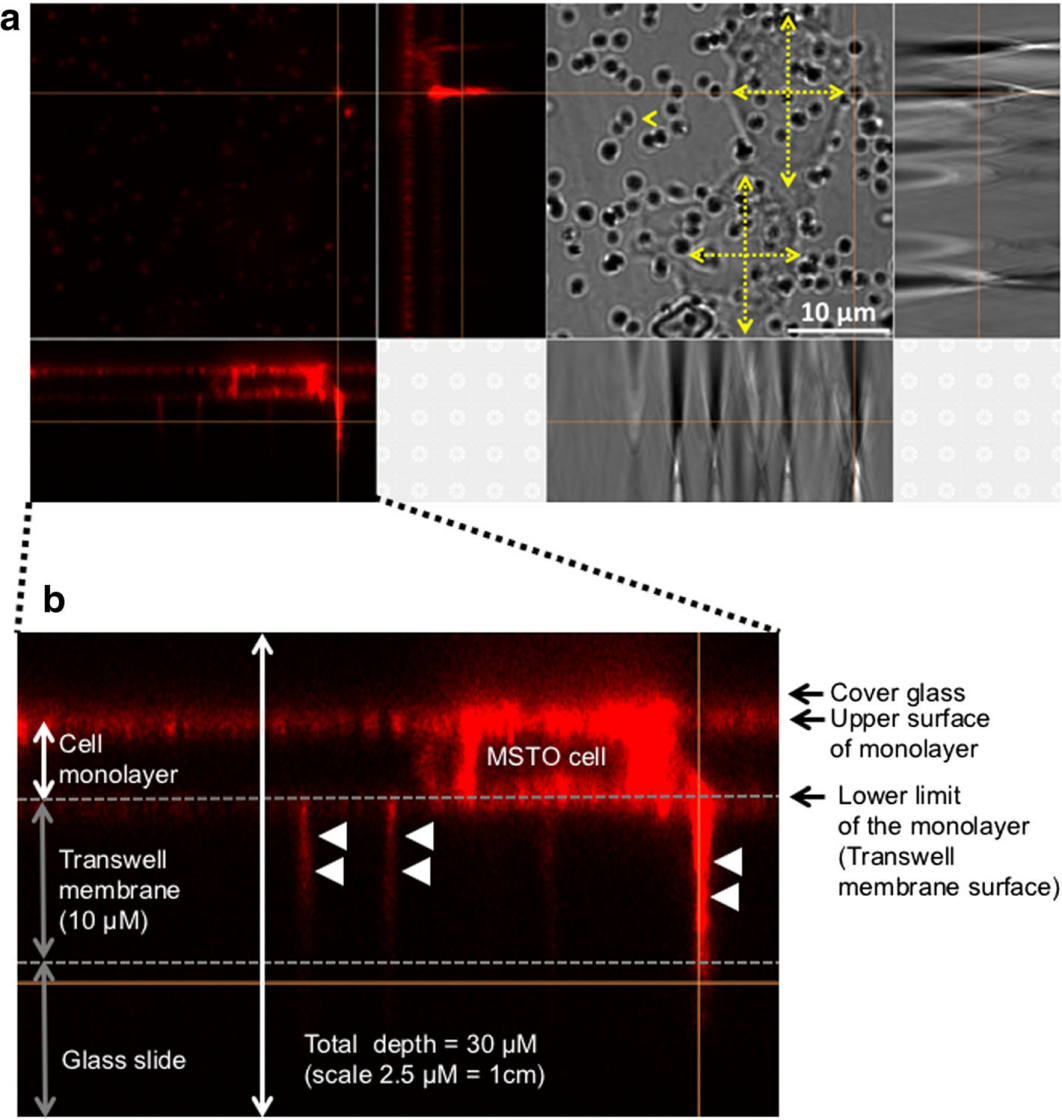
Confocal microscopy demonstrating TNTs traversing the pores of a transwell polyester membrane filter. MSTO cells were primed in TNT medium (low-serum, hyperglycemic medium) for 7 days prior to plating. To reduce exosomal trafficking, culture medium was removed 24 h prior to the experiment; cells were washed with PBS, and serum-free medium was added. Cells were stained with DiI prior to placement for culture on the top and bottom filters of a modified Boyden chamber encompassing a polyester filter containing pores measuring 400 nm in diameter. **a** DiI-stained cells with TNTs are shown on the left-hand panel. Comparison of MSTO cells and 400 nm pores is shown in the right-hand panel, with demonstration of Transmitted Detection (TD) imaging. Cells are marked by yellow cross bars and a pore is marked by an arrow head. **b** XZ view of Z-stacked confocal images of MSTO cells and associated TNTs/TNT-like structures crossing the membrane filter (indicated by arrowheads)

Our group has investigated formation and function of TNTs in a range of malignant and non-malignant cell lines, including pleural mesothelioma [27, 30, 32]. We have previously shown that these cell lines, including MSTO-211H (herein referred to as MSTO), readily form TNTs. Moreover, formation of TNTs is quantitatively upregulated when culturing these cells in low-serum, hyperglycemic culture media [27]; thus culturing cells in this medium could ‘prime’ cells to form TNTs and facilitate effective in vitro study. For those reasons, we selected MSTO cells as a model system for evaluation in the current study. As a control, we cultured MSTO cells in 6-well plates to confirm growth and formation of TNTs, and to provide representative examples of TNTs imaged using both light as well as fluorescent microscopic imaging in open culture (Fig. 1). To demonstrate TNT formation in the modified assay visually, we imaged TNTs protruding from cells and through the membrane filter as proof-of-concept that nanotubes were indeed forming and aligning with the pores as they extended toward the bottom chamber (Additional file 1: Figure S1 and Additional file 2: Figure S2). We removed and fixed the filter using paraformaldehyde and then performed confocal imaging. Three-dimensional confocal microscopy was performed to image TNTs in the z-plane (i.e. X-Z optical sectioned imaging), and y-planes (X-Y cross-sectional imaging for examination of depth). Here, we used both X-Z and X-Y plane stacking to image TNT-like structures passing through the transwell filter after 48 h of culture (Fig. 2a and b). Composite 3D images were constructed using Nikon AR software. To assess differences between cell diameters and pore sizes in the filter and to provide visual demonstration of TNT formation, we imaged the cancer cells overlying the pores and the TNTs traversing the entire span of the 10 μm polyester membrane (Fig. 2a). Our images detected a 30:1 ratio of greatest cell diameter to pore size, confirming that the cell bodies were too large to move from the upper chamber to the lower chambers via the pores (i.e. cell diameters of MSTO-211H cells were ~12 μm, and the pores were ~400 nm/0.4 μm, accounting for the 30-fold calculation). This confocal imaging experiment provided supportive evidence that TNTs (or at least TNT-like cellular extensions) can form and protrude through the length of the polyester membrane filter.

### Separation of cells using the transwell filter significantly reduces exosome trafficking by at least 66%, when quantified using nanoparticle tracking analysis

We next quantified differences in exosomal trafficking using the modified transwell assay as compared to open culture. Electron microscopy (EM) is the gold standard for visually identifying exosomes following isolation. They typically measure ~50–200 nm in diameter, and readily diffuse between cells in open culture. Several studies have demonstrated that physical barriers, such as the membranous filters used in transwell assays, can alone eliminate up to 85% of exosomal trafficking [20, 24]. Thus, we postulated that additional steps could either eliminate, or nearly eliminate, the remaining 15% of exosomes and thus prevent the effects of this transfer. The intent of excluding EV trafficking was to ensure that any remaining measures of intercellular transfer could be attributed to TNTs.

**Fig. 3:**
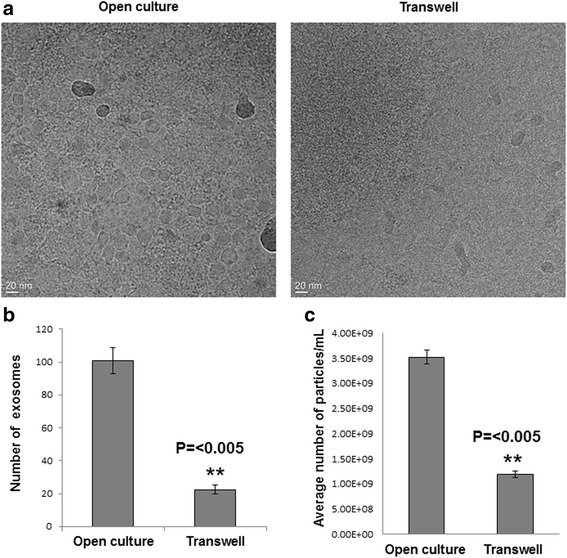
Transwell polyester membrane filters containing 400 nm-sized pores form a physical barrier that significantly reduces transfer of exosomes in the transwell assay. **a** Cryo-transmission electron microscopic (TEM) examination of exosomal transfer across a transwell assay membrane filter. TEM was performed on exosomes isolated in open culture wells (positive control, left) and the bottom transwell chamber (right) after 48 h of culture in serum-free media using the modifications described. **b** Quantification of exosomes transmitted to the bottom well of transwell chamber experiments, compared to exosomes in the open culture control. Exosomes were counted from 3 representative images per experiment and averaged. The relative reduction of exosomal trafficking using this transwell filter was ~ 80%, when assessed by using this method. **c** Nanoparticle tracking analysis of exosomes from above mentioned transwell and open culture experiments, quantifying the relative reduction at 66%. For statistical analysis, Student’s t-test was conducted, with a *p*-value of ≤0.05

Thus, our next step was to confirm the extent by which the transmembrane acted as a physical barrier to prevent EV trafficking of MSTO cells. To accomplish this, an equal number of MSTO cells (0.3 × 10^6^) was added to both the top and bottom chambers of the transwell assay (i.e. onto the membrane filter containing 400 nm/0.4 μm pores). In addition, we also added a predetermined number (2 × 10^9^) of exosomes (validated by Nanoparticle Tracking Analysis) to the cells in the top transwell chamber. After a 48-h incubation, exosomes isolated from the lower well were imaged to determine how many had passed through the porous membrane filter. As a positive control, a known number of exosomes (2 × 10^9^) were added directly to 6-well open cultures with MSTO cells and incubated for 48 h. Both experiments were carried out in serum-free mTeSR1 basal medium to ensure that exogenous exosomes were not added. Exosome fractions were isolated from open culture and the bottom transwell chamber, visualized by EM, and counted for comparison. Using this method, we determined that there were significantly fewer exosomes (80%) in the bottom transwell chamber than in open culture (*P* value <0.005) (Fig. 3b, lower-left). For more details on the experimental approach, please see the Materials and Methods section.

We employed nanoparticle tracking analysis (NTA) to more accurately quantify exosomes and MVs in our studies [35–37]. NTA is a highly sensitive method that utilizes the phenomenon that diffusivity of nanoparticles by Brownian motion in a liquid suspension is determined by size, temperature, and viscosity of the liquid in which they are contained. For this study, we used NTA to assess exosome concentrations more accurately than could be achieved using EM alone. Particles undergoing Brownian motion were digitally recorded; and their speed of motion was subjected to software-based analysis to determine the particle count and size. These findings demonstrated that the use of a porous filter containing the smallest pore sizes (400 nm) decreased trafficking of exosomes by ~ 66% (Fig. 3c, lower-right) (*p*-value <0.005). Specifically, as depicted in this figure, the average number of exosome particles/ml quantified from open culture was 3.52 × 10^9^, whereas the number of particles/ml detected in our transwell system was 1.19 × 10^9^. The percent difference was a 66.2% reduction: [(3.52 × 10^9^–1.19 × 10^9^)/3.52 × 10^9^] = 0.662. We noted that that this reduction was less than the 80% reduction we calculated by assessment using the less specific method of quantifying cryoEM-visualized exosomes. The more specific method of NTA provided a more feasible and likely more accurate platform for quantifying exosomes in this set of experiments.

**Fig. 4:**
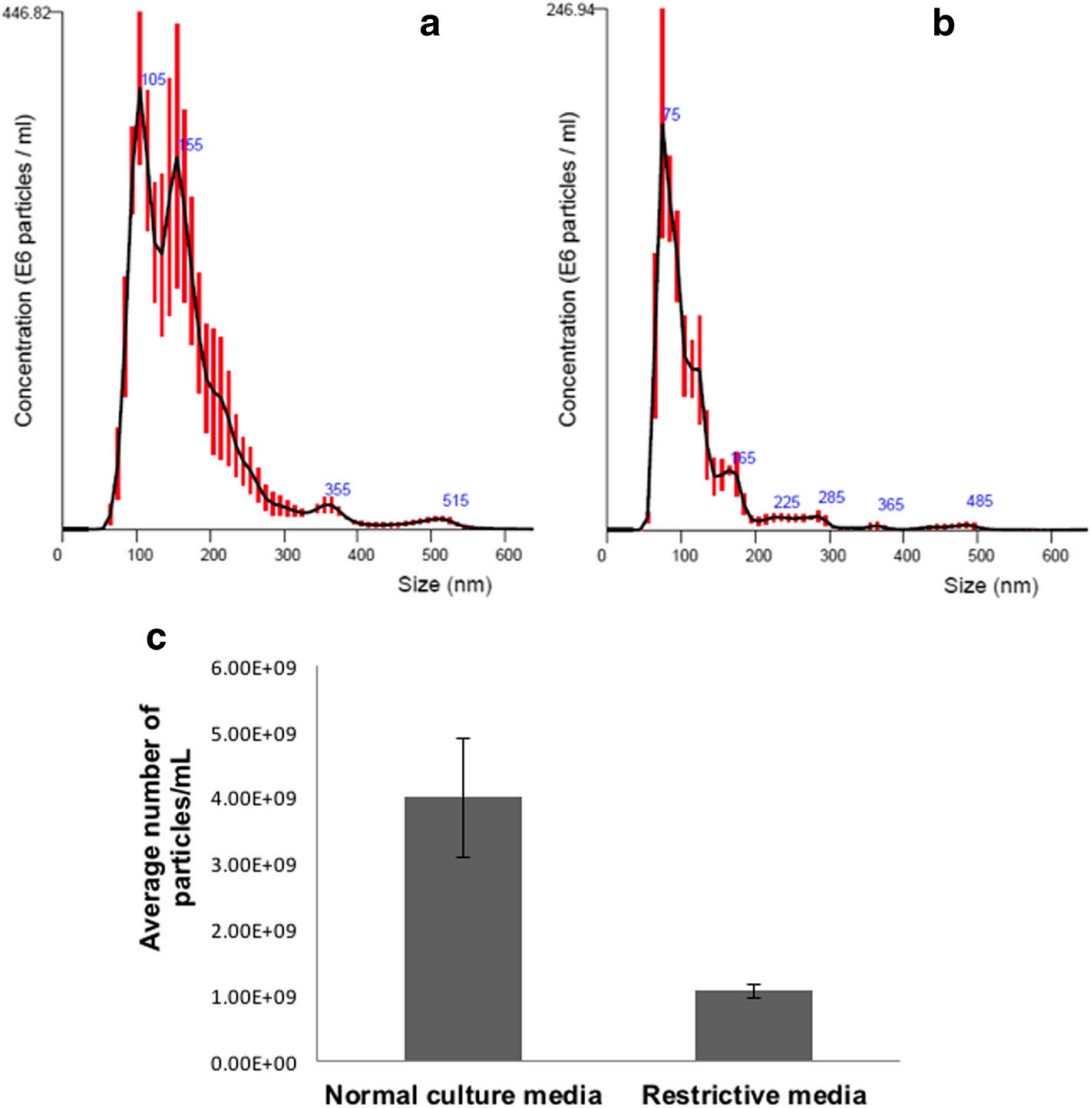
Modifications to culture conditions can further reduce exosome carryover to the lower transwell chamber, beyond the physical barrier provided by the transmembrane filter. “Restrictive media” conditions refer to washing cells and adding basal, serum-free (mTeSR) medium to avoid addition of exogenous serum-based exosomes. Nanoparticle Tracking Analysis (NTA) was used to determine the concentration of exosomes isolated following usual culture and passage conditions (**a**) as compared to concentration of exosomes isolated from cells subjected to restrictive medium conditions (**b**). Mean values from 5 independent runs are shown ± SD. The restrictive conditions reduced exosome contamination by ~75% compared to cells in normal culture medium conditions (**c**)

Having used NTA to confirm that the porous membrane filter significantly reduced exosomal trafficking, we next sought to exclude any trafficking or effects of the remaining 20% of EVs. To do this, we cultured cells in conditions designed to reduce effects of exosome contamination, secretion, and uptake prior to the transwell assay. We washed MTSO cells and then added culture medium containing exosome-depleted serum. MTSO cells cultured in “restrictive” conditions (i.e. with the stated modifications) or usual passage media (10% FBS at 37 °C) were added to the top chamber of separate transwell plates. Cells were then incubated for 48 h prior to isolating exosomes that passed through the membrane filter (400 nm-sized pores) into the lower well of the transwell chambers. We then again used NTA to quantify the concentration of exosomes. We found that a greater concentration of EVs was detected among cells cultured in usual passage medium (Fig. 4a) than among cells cultured under modified “restrictive conditions” (Fig. 4b). Specifically, comparison of the NTA profiles revealed that the use of exosome-restrictive conditions independently decreased overall exosomal carryover by ~74% (i.e. in addition to the 66% reduction already seen by using the transwell membrane filter) (Fig. 4c). Specifically, as depicted in this figure, the average number of exosome particles/ml quantified from normal culture medium was 4.00 × 10^9^, whereas the number of particles/ml detected in our restrictive conditions was 1.06 × 10^9^. This percent difference was a ~73.5% reduction (rounded up to 74%): [(4.00 × 10^9^–1.06 × 10^9^)/4.00 × 10^9^] = 0.735. From these data, we determined that a 74% reduction of EVs not eliminated by the membrane filter alone resulted in a net, or overall, reduction in EV trafficking of 95% [(66% + (74% of 20%)) = 0.8 + 0.148 = 0.948 (rounded up to 95%)]. From these data, we concluded that the addition of the specified steps to modify the standard transwell chamber system allowed us to eliminate 95% of all EV trafficking between cells separated by the filter, thus allowing for more selective communication to take place via an alternate route, TNTs.

As a quality measure of our experimental approach in exosome recovery, we confirmed that our technique was accurate and effective. To accomplish this, we added a known amount of exosomes (2 × 10^9^) determined by NTA to 6-well plates containing no cells. We collected the media after 48 h, isolated the exosomes, and quantitated the EVs by NTA. The efficiency of exosome recovery was ~ 94%, thus providing assurance that our technique of exosome recovery was sufficient (Additional file 3: Figure S3).

### Assessment of trafficking of exogenously added exosomes: The transwell polyester membrane containing 400 nm-sized pores serves as an effective physical barrier to exosomal trafficking as compared to open culture without barriers separating cells

**Fig. 5:**
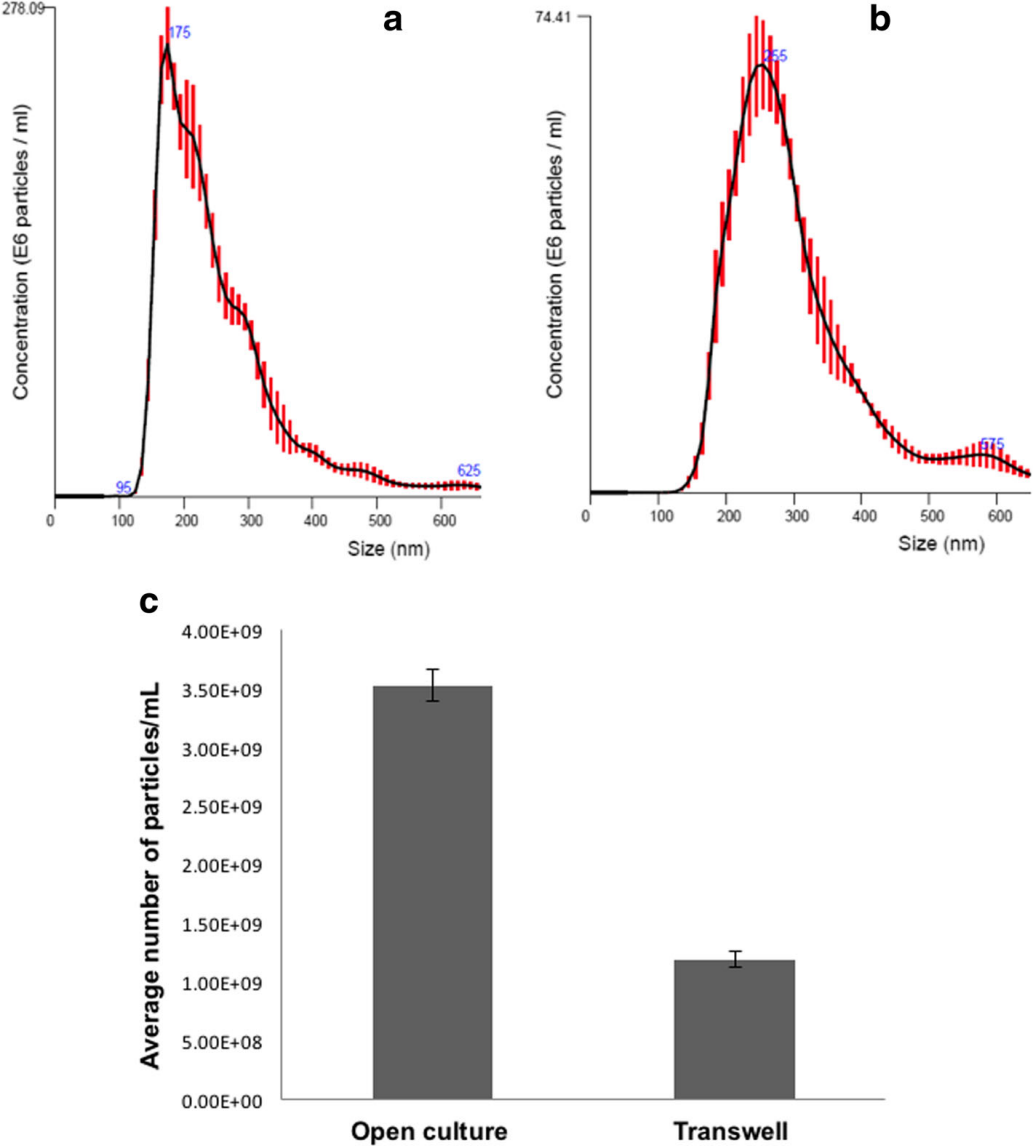
Assessment of trafficking of exogenously added exosomes: the transwell polyester membrane containing 400 nm-sized pores serves as an effective physical barrier to exosomal trafficking as compared to open culture without barriers separating cells. MSTO cells were prepared under restrictive conditions (using exosome-depleted serum); and then plated and incubated with exogenously added VAMT-derived exosomes (2 × 10^9^) in triplicate for 48 h. After incubation, medium from the bottom chamber was collected and subjected to exosome isolation and NTA as explained in the Methods section. NTA-assessed exosome concentrations (reported as number of particles/ml × 10^6^) after open culture in 6-well plates (**a**); and after culture in the Boyden chamber transwell experiments (**b**). In the latter experiment, VAMT-derived exosomes were added to the top of the transwell chamber, and medium was recovered after 48 h from the lower chamber for NTA. The transwell filter with 400 nm pore size can significantly block exosome transfer, independently of other steps (**c**). Mean values from 5 independent runs are shown ± SD

The use of NTA was straightforward and allowed us to quantify EVs with greater accuracy than by using visual inspection and counting via EM. Thus, repeat studies were carried out to more accurately assess the ability of the membrane filter to reduce EV trafficking. We next carried out additional experiments using either open cultures or transwell chambers under the modified ‘restrictive conditions’. We added exosomes to MSTO cells, incubated them for 48 h, then recovered exosomes and analyzed them using NTA (Fig. 5a showing data for open culture; and Fig. 5b showing data for modified the transwell approach). Comparison of these two profiles revealed that the media recovered from the transwell chamber in which exosomes were separated by a physical barrier contained significantly fewer exosomes (a 70% reduction) (Fig. 5c).

### Pharmacologic blockade of EV uptake by putative recipient cells: A final step to preventing downstream effects of EV trafficking

**Fig. 6:**
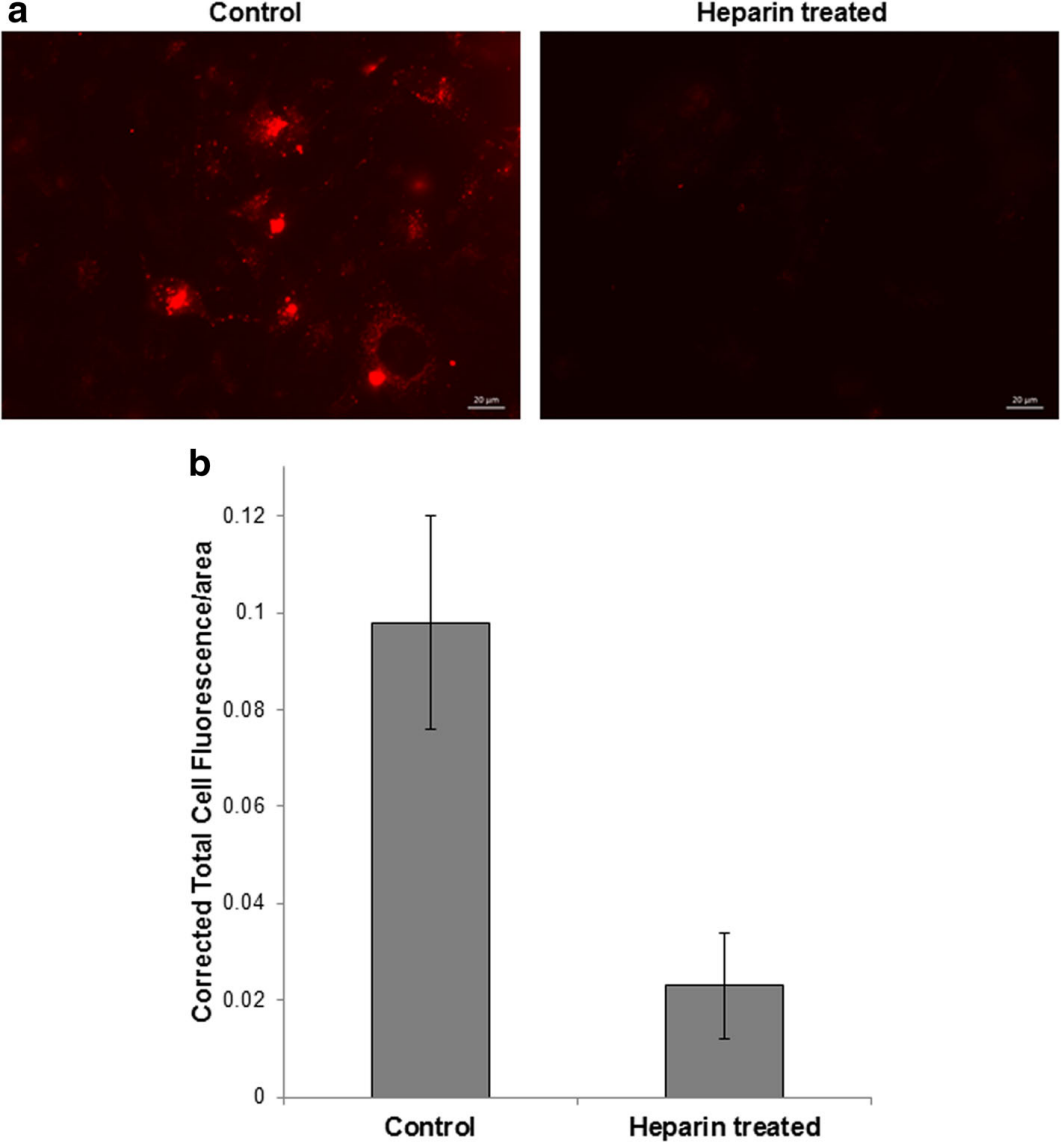
Exosome uptake is effectively blocked by pharmacologic treatment with heparin. **a** PKH26 (red dye)-labeled VAMT-derived exosomes were added to MSTO cells on the top of the transwell/Boyden chamber; and to the lower chamber, we added MSTO cells pre-treated with or without 10 μg/mL heparin. Extent of exosomal uptake of cells in the lower chamber was analyzed after 24 h for both conditions (scale bar = 20 μm). The reduction in red fluorescence in heparin pre-treated cells indicates efficient blocking of exosome uptake. **b** This experiment was performed in triplicate; representative images were taken from each replicate and subsequently used for calculations to compare the Corrected Total Cell Fluorescence (CTCF) per area of the control and heparin-treated cells. CTCF/area was calculated for 36 cells in the control group, and 72 cells in the heparin-treated group, and the results were averaged. Our results demonstrate that heparin treatment of recipient cells can significantly block uptake of the majority of remaining exosomes. Mean values are shown with ± standard error. *p*-value = 0.04

We hypothesized that the final step in preventing EV-mediated intercellular communication would be to block exosomal uptake by recipient cells in the lower transwell chamber. Thus, we selected heparin as a pharmacologic inhibitor of exosomal uptake, again using a membrane filter containing 400 nm-sized pores and restrictive culture conditions. Heparin has been shown to be effective at reducing exosome uptake at a concentration of 10 μg/ml [31, 38–40]. MSTO cells were pre-treated at this dose for 30 min per prior protocols [31, 38, 40] before being washed and added to the lower transwell chamber. MTSO cells and VAMT mesothelioma-derived exosomes stained with red fluorescing PKH26 dye were added to the top transwell chamber for a 15-min incubation; cells were then washed in order to remove any remaining unbound or free-floating exosomes. After 24 h of incubation, we used fluorescence microscopy to determine the amount of exosome uptake in recipient cells in the lower chamber that were treated with heparin; these results were compared to cells that had not been pretreated with heparin (Fig. 6a; Additional file 4: Figure S4). Fluorescence microscopy images were analyzed using Image J by determining the Corrected Total Cell Fluorescence (CTCF), and divided by cell area to calculate CTCF/area, a technique that we have reported previously [30]. Heparin treatment of cells in the lower chamber reduced the CTCF/area by 76.5% as compared to non heparin-treated cells [(control 0.098 – heparin 0.023)/0.098 = 0.765 = 76.5% decrease; *p*-value = 0.04] (Fig. 6b). Although CTCF/area is not an exact surrogate for quantification of EVs, this significant decrease supports the notion that pharmacologic blockade with heparin likely blocks nearly all of the remaining 5% of EVs that remain following the use of each of the prior steps we have outlined.

### Scanning electron microscopy demonstrates TNT-like structures extending through the 400 nm pores

**Fig. 7:**
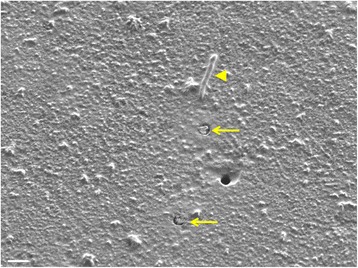
Scanning Electron Micrograph (SEM) of TNT-like structures penetrating the 400 nm pores of the transwell membrane. MSTO cells were grown on the transwell membrane culture inserts for 48 h and then fixed using PFA. On the bottom of the membrane, TNT-like structures were identified and imaged without staining. The image provides evidence that TNTs have the capacity to penetrate and traverse the transwell membrane via the 400 nm-sized pores of the polyester filter (scale bar 1000 nm). that formed through the membrane pores, and extended beyond the membrane are shown, marked by arrowheads. Several TNTs were disrupted by the fixation process, but are shown in cross-section (arrows)

The transwell membrane apparatus has been extensively used to assess invasive capacity and migration of cancer cells. The finding of long membrane extensions penetrating from cells cultured in a top chamber toward the bottom chamber through a 1000 nm pore size filter has been reported using HCT-116 colon cancer cells [41]. To address the issue of whether TNTs can penetrate through the transwell membrane of 400 nm, we preconditioned (primed) MSTO cells in TNT media for a week and plated them onto transwell inserts containing polyester membranes with 400 nm pores. After 48 h, the membranes were removed, washed, and fixed with 4% PFA; and the filters mounted onto a scanning electron microscopy (SEM) stage, thereby exposing the bottom side of the membrane for imaging. Imaging revealed TNTs penetrating through the 400 nm pores and penetrating the opposite side (Fig. 7). Quite interestingly, we also observed several TNTs that appeared to have fractured in the fixation process, revealing the interior of TNTs exposed in cross-section (Fig. 7 and Additional file 5: Figure S5).

### An example of how this assay can be used to assess TNT-selective intercellular communication

Our group recently reported the use of this modified transwell method for assessing TNT-mediated bystander effect following oncolytic viral infection [33]. In that study, the transwell membrane acted as a physical barrier that eliminated gap junction-mediated cell-to-cell spread of a viral thymidine-kinase activated nucleoside analog compound (ganciclovir) that is often used to study the bystander effect. Use of the actin-destabilizing agent Cytochalasin B, which prevents and disrupts TNT formation, abrogated the bystander effect in this in vitro system, providing further support for the utility of this modified assay.

## Discussion

In our studies of cancer TNTs, we have modified standard commercially-available transwell assays and adapted additional experimental protocols to minimize contamination by exosomes, microvesicles, and microparticles. First, our prior work demonstrated that “priming” cells using low-serum, hyperglycemic medium (“TNT medium”) stimulated and quantitatively increased TNT formation, promoted in vivo tumor growth, and altered transcription of key regulatory factors [42]. We therefore primed cells for up to 7 days in TNT medium to increase the likelihood of TNT formation. Second, to decrease the number of exosomes added to the top well at the beginning of experiments, culture medium was removed 24 h prior to the experiment; cells were washed with PBS, and serum-free medium was added. We used mTeSR1 minimal medium that contained bovine pituitary extract and epidermal growth factor (EGF), but no serum. Third, we used a membrane filter with the smallest commercially available pore size (400 nm) to ensure that it not only served as an effective barrier to cellular mobility, but also as a conduit for TNT formation. In addition, we used confocal microscopy to image cells overlying the membrane in the top chamber and confirmed that the cell diameter of a malignant cell line (MSTO-211H) was ~ 12 μm (i.e. 30 times larger than the diameter of individual 400 nm pores). Fourth, to mitigate potential effects of exosomal uptake by recipient cells in the bottom chamber, we used pharmacologic agents that are known to prevent this uptake. Examples of such agents include heparin, GW869 (a sphingomyelinase inhibitor), ammonium chloride, and chloroquine [25, 38–40, 43]. For the present study, we opted to use heparin to prevent exosome uptake by recipient cells in the bottom chamber of the assay due to supportive evidence in the literature, as well as its widespread commercial availability. Our data validated this cumulative series of modifications for studying TNT-specific communication in a transwell assay.

Many studies in the field of intercellular communication have largely focused on identification of the actual cargo rather than specifying the exact intercellular route, or routes, of transport. To accurately determine the role of TNTs in cellular communication, it will be critical to accurately assess TNT-specific transport as compared to intercellular transport occurring via other means. Cells in immediate proximity are most likely to communicate via gap junctions. Long-distance communication in vitro can occur via diffusible lipophilic carriers (e.g. exosomes, MVs). The presence of EVs can potentially confound experiments designed to investigate the effects of TNT-specific intercellular transfer of cargo. For this reason, validated assays are needed to differentiate the effects of TNT-mediated transfer from transfer occurring via EVs.

To date, there has been no assay universally used to confirm TNT-specific transport. The assay most commonly described in the literature is the classic transwell assay, in which a porous membrane filter is used to separate two populations of cells. The membrane filter in most commercially available transwell assays measures ~ 10 μm in thickness, thus obstructing cell-to-cell contact and subsequent transfer via gap junctions. At least two studies have demonstrated that use of a transwell assay can reduce exosomal traffic (ie, contact-independent) communication by nearly 85% [20, 24]; however, in other studies, a similar transwell system has been used with the intention of examining exosome-specific communication [25]. We believe that previous protocols can be consolidated but do need to be reviewed critically to ensure that the experimental approaches are adequate for addressing questions at hand. The transwell assay/Boyden chamber system has been variably used as a negative control. This concept presumed, however, that the porous membrane filter would wholly prevent TNT formation and cargo transfer, or as a means to in fact demonstrate TNT-mediated communication but without exclusion of EV (Summarized in Tables 1 and 2). In addition to transwell assays, open co-culture approaches followed by cell sorting have been used to present claims of cell-cell transfer in cells shown separately to form TNTs; however, this experimental approach cannot discriminate TNT-mediated transfer from other forms of cargo transfer, including gap junctions, EV, and free-floating cargo in culture media. In our current study, we used confocal imaging to demonstrate that TNTs (or TNT-like protrusions) can in fact form effectively in the context of a transwell chamber, and that validated measures can be taken to markedly eliminate EV trafficking, which would otherwise confound TNT-communication specific studies.

Our studies confirm findings from other researchers that a transwell membrane filter can serve as an effective physical barrier to reduce EV trafficking. However, we also considered that the same membrane would likely reduce TNT formation at least to some extent. Nonetheless, while the overall number of TNTs might be reduced by this physical barrier, TNTs can, in fact, form by penetrating the barriers through the open nano-sized pores. In a recently published study from our group, we used this modified transwell assay to assess the ability of TNTs to propagate the bystander effect for inducing cytotoxicity in connected cancer cells following infection with an oncolytic herpes simplex virus-1 (HSV-1) [33]. We found that TNTs were in fact able to mediate the bystander effect following addition of a nucleoside analog (ganciclovir) that was activated by HSV-1 viral thymidine kinase, and that propagation of this activated drugs via TNTs led to a synergistic increase in cell death. These results verified that our modifications can be used to assess TNT-specific intercellular communication, and provide one example of they can be used in functional assessments of TNTs.

The steps outlined here represent a modified form of an assay that is widely available commercially, and thus available to any interested researchers. These steps are just one attempt to exclude the influence of non-TNT-based communication. The study was designed to establish an “assay of exclusion” that ideally would leave only TNT-mediated communication remaining. We note that, based on prior studies and the current study, that the membrane filter can prevent transfer of up to 85% of extracellular vesicles. Thus it is also likely that the same filter would severely diminish TNT formation quantitatively. We performed confocal fluorescence microscopy in an effort to confirm visually whether any TNTs could traverse the membrane filter at all despite this porous barrier. Certainly the physical set-up of the assay presented a high level of technical difficulty in carrying this out, but we postulated that the pores could potentially permit formation of at least some TNTs from the cells in the top chamber. While in most studies TNTs are imaged in vitro in the XY plane, our efforts here included z-stacked imaging in the XZ plane to confront the 3-dimensional aspect of the two-chamber vertical transwell apparatus. Our images demonstrated long fluorescent TNT-like structures traversing the filter. We entertained the possibility whether this finding actually represented transmission of light through the 400 nm pores, rather than actual physical structures. However, we only visualized several of these protrusion-like extensions, whereas there were dozens of pores seen in each visual field (as demonstrated in Fig. 2). We concluded that our confocal microscopy approach would have shown indiscriminate passage of light through the pores had that been the case.

The relatively small number of TNT-like protrusions extending through the membrane, as compared to the high number of membrane pores, when taken in combination with further direct imaging of protrusions via EM extending outward from the undersurface of the membrane (Fig. 7), further supports the notion that these are TNT-like structures. Interestingly, in addition to visualizing intact protrusions using this EM technique, we did observe several protrusion-like extensions that appeared to have “broken off” during the fixation and handling process. We speculate that this finding provides a glimpse at the interior of TNTs through a cross-sectional view. For comparison, an open filter pore (seen in the lower right-hand quadrant of Fig. 7) appears black, whereas the two pores indicated by yellow arrows show what appear to be groups of filaments that most likely represent filamentous actin. It is well-established that TNTs are actin-based cellular extensions, and that at times based on whether they are protruding or receding, they also may contain dynamic microtubules. To our knowledge, few groups have been able to image TNTs in cross-section. Mineo et al. provided striking cross-sectional and longitudinal examination of TNTs to effectively demonstrate TNT-mediated transport of exosomes between chronic myelogenous leukemia cells using 3-dimensional reconstruction of confocal images [44]. In one of our prior studies, scanning EM was able to elucidate TNTs forming between MSTO cells in sagittal/longitudinal section, also suggesting bundling of filaments within TNTs [30]. As the mechanism(s) of TNT formation remains an area of intense study, this accidental finding of cross-sectional views of TNTs using the transwell filters could potentially be harnessed to more clearly define the underlying key structure/function components of TNTs.

We combined several methods with the overarching goal of significantly reducing, if not altogether eliminating, trafficking of exosomes and MVs. We found that NTA was a highly useful and accurate method for assessing exosomal/MV concentration; and thus, we used NTA extensively to quantify relative differences in concentration with or without interventions. EM and fluorescence activated cell sorting (FACS) are the most commonly used tools for exosome analysis. The EM approach has disadvantages, as it can be a time-consuming and labor-intensive process that is further limited by potential for artifacts generated during sample preparation. Furthermore, a potential limitation of FACS is its relative insensitivity for particles less than 500 nM in diameter [35, 45]; and this is especially true for particles that are less than the anticipated width of 50–200 nm for most exosomes and MVs. More recently, NTA has been suggested as a reliable method of exosome quantification [35, 37]. In the current study we showed that NTA can be successfully and reliably employed for the analysis of exosomes in routine laboratory situations. Advantages of this technique include rapid and efficient use, and potentially less costly as compared to enzyme-based assays and other imaging methods.

The approach outlined herein, as with any assay, has potential methodological and functional limitations, as discussed. For the field of TNT biology to move forward, a variety of assays will need to be designed and appropriately validated to answer key specific questions, including the question of functional effects of TNT-specific transfer. It is our hope that our efforts will be among many that will be validated and used to study TNTs in the years to come.

## Conclusions

The field of TNT biology and intercellular communication has advanced from an in vitro study to that of in vivo relevance to human diseases. TNTs are actively being investigated for their potential role in a number of human diseases, including cancer, blood-based disorders, neurologic and inflammatory diseases. TNTs are a natural biologic conduit for intercellular signaling and transport of cellular cargo. Recent advances in understanding the function and mechanism(s) of TNTs challenge the paradigm that gap junctions, exosomes, or cytokines, and other diffusible chemical signals are exclusive modes by which cells communicate. Thus, the method described here for evaluating TNTs can be applied to improve our understanding of their role in multiple physiologic processes and types of disease. As the nascent field continues to grow, validated assays for studying TNT function and effects in vitro will be critical for differentiating this form of cellular transport from other modes of communication.

## Additional files

Additional file 1: Figure S1. Schematic diagram of the transwell experiment. DiI-stained cells were placed on the polyester membrane on the transwell insert and allowed to incubate to permit TNT formation, as described in the Methods section. (TIFF 1454 kb)

Additional file 2: Figure S2. 3-dimensional confocal fluorescent imaging demonstrates TNTs/TNT-like extensions forming and protruding through the porous transmembrane filter. A portion of the polyester membrane along with cells was cut from the transwell, mounted on a glass slide with a cover glass, and analyzed by confocal microscopy. (TIFF 1375 kb)

Additional file 3: Figure S3. Validation of exosome recovery. VAMT exosomes (2 × 10^9^) were added into 6- well plates containing 2 ml of serum free basal mTeSR1 medium and incubated for 48 h. After 48 h, medium was collected and subjected to exosome isolation for NTA. Almost all of the 2 × 10^9^ exosomes added were recovered without a significant loss, with a recovery efficiency of >95%. The exosome samples were run 5 times and averaged. SD is shown as the error bar. (TIFF 1107 kb)

Additional file 4: Figure S4. Uptake of exosomes crossing the transwell membrane is significantly decreased by heparin treatment of recipient cells. PKH26 (Red) labelled VAMT exosomes were added to MSTO cells pre-treated with (b) or without (a) 10 μg/mL heparin. Exosome uptake was analyzed after 24 h of culture. DIC and DIC + fluorescent merged images of control and heparin-treated cells are shown. (TIFF 2404 kb)

Additional file 5: Figure S5. Scanning Electron Micrograph (SEM) of TNT-like protrusions emerging on the other side of the transwell membrane. This image provides supporting evidence that TNTs have the capacity to penetrate the pores of the transwell membrane. We also noted the presence of broken TNTs in the pores exposing them in cross-section; we postulate that this occurred due to the structurally sensitive nature of TNTs and to the high negative pressure during SEM imaging. Broken TNTs are marked by arrows. (TIFF 2554 kb)

## Abbreviations

CTCF: Corrected total cell fluorescence
EM: Electron microscopy
EV: Extracellular vesicles
FACS: Fluorescence activated cell sorting
MV: microvesicles
nm: nanometers
NTA: Nanoparticle tracking analysis
PBS: Phosphate buffered saline
TEM: Transmission electron microscopy
TNTs: tunneling nanotubes
μm: microns
