# Supplementary figures and images for: A transwell assay that excludes exosomes for assessment of tunneling nanotube-mediated intercellular communication
Source: Cell Commun Signal. 2017 Nov 13;15:46. doi: 10.1186/s12964-017-0201-2 (PMC5683209; doi:10.1186/s12964-017-0201-2)

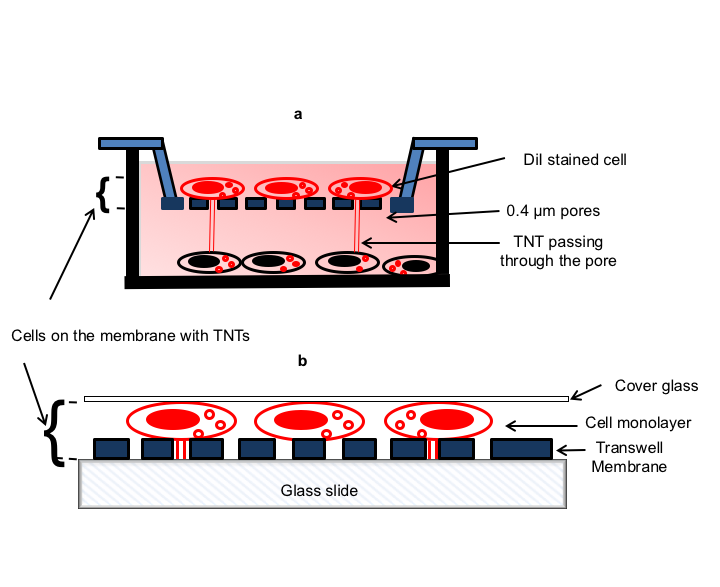

Supplement: Supplementary file 1 — Schematic diagram of the transwell experiment. DiI-stained cells were placed on the polyester membrane on the transwell insert and allowed to incubate to permit TNT formation, as described in the Methods section. (TIFF 1454 kb) [file 12964_2017_201_MOESM1_ESM.tif]

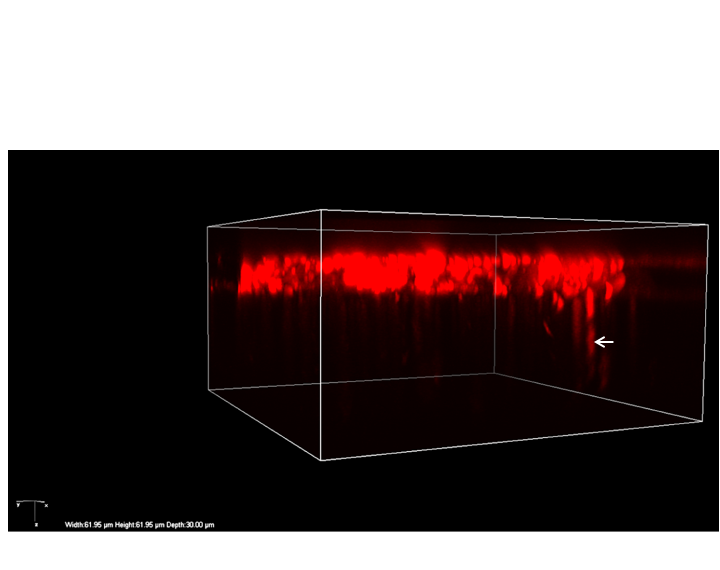

Supplement: Supplementary file 2 — 3-dimensional confocal fluorescent imaging demonstrates TNTs/TNT-like extensions forming and protruding through the porous transmembrane filter. A portion of the polyester membrane along with cells was cut from the transwell, mounted on a glass slide with a cover glass, and analyzed by confocal microscopy. (TIFF 1375 kb) [file 12964_2017_201_MOESM2_ESM.tif]

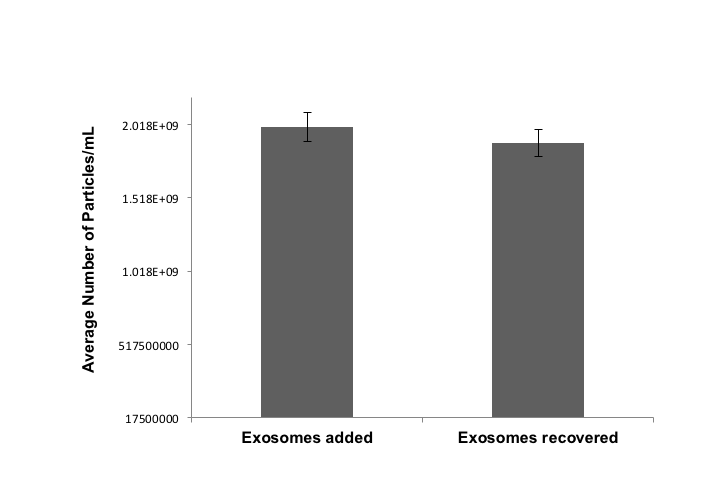

Supplement: Supplementary file 3 — Validation of exosome recovery. VAMT exosomes (2 × 10^9^) were added into 6- well plates containing 2 ml of serum free basal mTeSR1 medium and incubated for 48 h. After 48 h, medium was collected and subjected to exosome isolation for NTA. Almost all of the 2 × 10^9^ exosomes added were recovered without a significant loss, with a recovery efficiency of >95%. The exosome samples were run 5 times and averaged. SD is shown as the error bar. (TIFF 1107 kb) [file 12964_2017_201_MOESM3_ESM.tif]

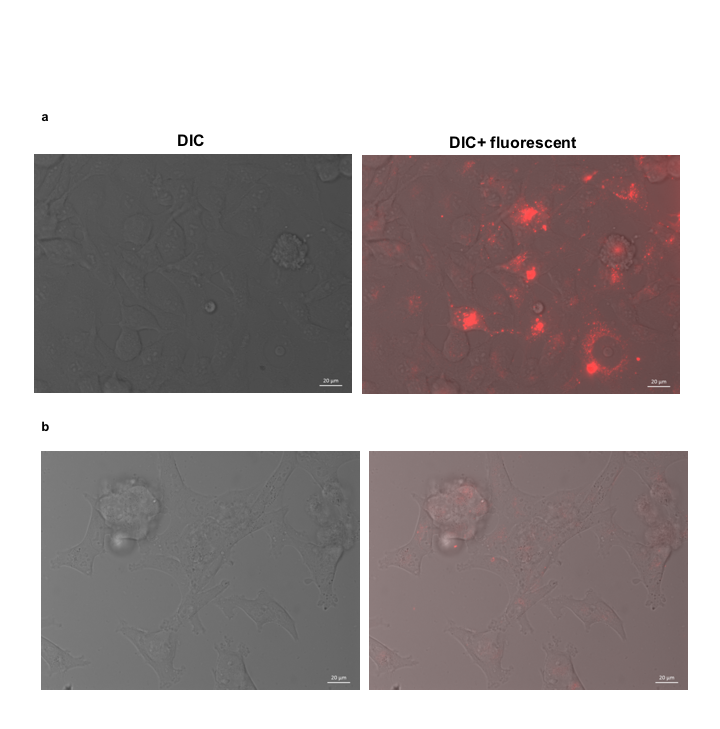

Supplement: Supplementary file 4 — Uptake of exosomes crossing the transwell membrane is significantly decreased by heparin treatment of recipient cells. PKH26 (Red) labelled VAMT exosomes were added to MSTO cells pre-treated with (b) or without (a) 10 μg/mL heparin. Exosome uptake was analyzed after 24 h of culture. DIC and DIC + fluorescent merged images of control and heparin-treated cells are shown. (TIFF 2404 kb) [file 12964_2017_201_MOESM4_ESM.tif]

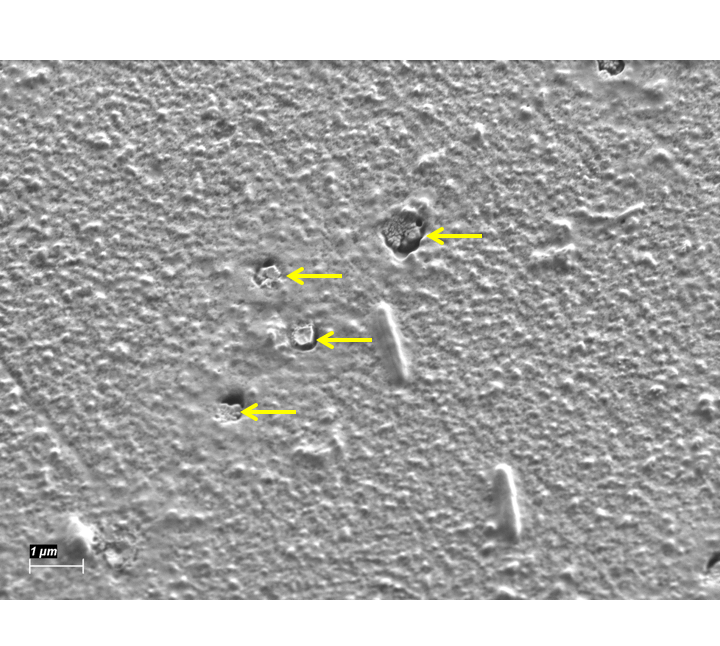

Supplement: Supplementary file 5 — Scanning Electron Micrograph (SEM) of TNT-like protrusions emerging on the other side of the transwell membrane. This image provides supporting evidence that TNTs have the capacity to penetrate the pores of the transwell membrane. We also noted the presence of broken TNTs in the pores exposing them in cross-section; we postulate that this occurred due to the structurally sensitive nature of TNTs and to the high negative pressure during SEM imaging. Broken TNTs are marked by arrows. (TIFF 2554 kb) [file 12964_2017_201_MOESM5_ESM.tif]
